# External Developmental Assets and Positive Identity Among Emerging Adults in Norway, Romania, Slovenia, and Turkey

**DOI:** 10.3389/fpsyg.2021.656972

**Published:** 2021-07-14

**Authors:** Ayfer Dost-Gözkan, Ana Kozina, Delia Stefenel, Nora Wiium

**Affiliations:** ^1^Department of Psychology, Ozyegin University, Istanbul, Turkey; ^2^Educational Research Institute, Ljubljana, Slovenia; ^3^Faculty of Social and Human Sciences, Lucian Blaga University of Sibiu, Sibiu, Romania; ^4^Department of Psychosocial Science, Faculty of Psychology, University of Bergen, Bergen, Norway

**Keywords:** developmental assets, positive youth development, emerging adulthood, positive identity, thriving, youth policies

## Abstract

The present study adopts The Developmental Assets and Positive Youth Development (PYD) perspectives which (in contrast to the deficit-based approaches which highlight risks and deficit in youth development) claim that young people have potencies to achieve optimal development if supported by their social environment. Extant research indicates that developmental assets are linked with a variety of thriving indicators. The present research aimed to contribute to the PYD research by examining the external developmental assets (support, empowerment, and boundaries and expectations) emerging adults (*N* = 2055; age range = 18–28) perceived in their social environment and the level of their positive identity in four countries (Norway, Romania, Slovenia and Turkey), which have different rankings in economic, human, and youth development indices. The present study also tested a path model, which examines the links between external development assets and positive identity. Findings indicated that although there are differences in the reports of external developmental assets and positive identity, external assets and positive identity are similarly and positively linked across the four countries. The findings build on the existing literature by showing that developmental assets are conducive to positive outcomes cross-nationally despite country-level differences in the experience of external assets. Policy implications of the findings were discussed from the perspective of ecological theory as well as Developmental Assets and Positive Youth Development Frameworks.

## Introduction

The World Health Organization (WHO) defines health as ‘a state of complete physical, mental and social well-being and not merely the absence of disease or infirmity’ (World Health Organization (WHO), 1946, p. 1). WHO also states that enjoyment of the highest standard of health is one of the fundamental rights of individuals. The emphasis that the absence of disease is not a sufficient condition for being considered healthy and that health requires physical, mental and social well-being, invites more preventive approaches and promotion of well-being from an asset-based perspective, where resources and opportunities are offered. In the field of philosophy of law and human rights, the Capability Approach (Nussbaum, 2003; Sen, 2005) puts forward a strong argument, which does not only endorse an asset-based approach to human development, but it also highlights that facilitating the development of everyone’s capabilities is the responsibility of the social and legal system.

In the field of youth development, Developmental Assets Framework and Positive Youth Development (PYD) perspectives have been prominent in shifting the theoretical paradigm from deficit-based approaches to asset-based approaches. Deficit-based approaches foregrounds risks and problem behaviors. PYD perspective, on the contrary, assumes that young people have potencies that can serve for optimal development when environmental opportunities are provided (Porter, 2010). In line with the strength-based approaches to youth development, healthy adolescence has been defined in terms of positive developmental tasks or goals that adolescents are expected to achieve (e.g., Scales et al., 2000).

The present research is based on Developmental Assets and PYD Frameworks. Developmental assets have been framed as essential resources conducive to thriving (Benson, 2007; Search Institute, 2007). Developmental assets comprise two major resources: social-environmental resources (*external assets*) and personal resources (*internal assets*), each consisting of four subcategories of assets. External assets include *support* from the family, school, neighborhood, *empowerment* (e.g., feeling safe and secure, having roles and responsibilities and decision-making opportunities), *boundaries and expectations* (e.g., rules and boundaries set by the family, and the school, and expectations from the young person) and *constructive use of time* (e.g., engagement in creative activities). Internal assets include *commitment to learning* (e.g., valuing learning), *positive identity* (e.g., having a sense of purpose and having confidence), *positive values* (e.g., having integrity and responsibility) and *social competence* (e.g., efficient interaction with others, having adaptive and coping skills). Overall, these assets cover both the individual level resources and broader relational contexts, such as family, school, community, social network and peers (Scales et al., 2017), which are also termed as developmental relationships (Roehlkepartain et al., 2017). Although external assets indicate social environmental resources necessary for optimal development and thriving, internal assets may be seen as both developmental resources as well as health indicators (i.e., having a positive sense of self, endorsement of and commitment to positive values and learning, having social skills) which fits to the health definition of World Health Organization (WHO) (1946).

Adopting the perspectives of Developmental Assets and PYD, the present research aimed to examine the extent of availability of the four external assets, and the association between the external assets and positive identity (an internal asset and an indicator of well-being) among emerging adults across the four countries in Europe: Norway, Romania, Slovenia, and Turkey. Below research on developmental assets across the globe is reviewed and the rationale for conducting a study with a sample of emerging adults, and in four European countries with different social, cultural and economic background will be explained.

### Developmental Assets and Positive Youth Development Research Across the Globe

The Developmental Assets framework has been examined and shown to be valid across ethnically, socioeconomically, and culturally diverse groups (Scales et al., 2017). Research findings, however, have shown that many young people do not experience developmental assets at an optimal level. Data from developing countries, which includes mostly disadvantaged youth, has indicated that although both individual and environmental assets are associated with a wide range of well-being indicators, the number of young people who experience these assets is just above the vulnerable level. Data from developed countries do not show a more favorable picture. Considerable number of young people across developed countries have only moderate levels of developmental resources and those who enjoy high levels of assets is only a minority (Scales et al., 2016). There is also a downward developmental trend indicating that the experience of assets declines during adolescence (Scales et al., 2016, 2017; Soares et al., 2019). Researchers conclude that many adolescents across the world do not have the basic personal and environmental assets to achieve successful transition to adulthood (Scales et al., 2016).

Researchers, however, stress the importance of support and opportunities for the development of the skills necessary for thriving and for the promotion of human wellness (Scales et al., 2017). Theoreticians of human development strongly advocate that facilitating the development of individual capabilities (Nussbaum, 2003; Sen, 2005) and helping young people to achieve developmental transitions is the responsibility of societies (Scales et al., 2016). Search Institute’s Developmental Relationships Framework (Roehlkepartain et al., 2017) and research findings on the relationships between developmental assets and PYD also underlined the importance of supportive relationships (Bowers et al., 2015). Indeed, Benson (2010) powerfully states that after long years of extensive research, researchers and practitioners ended up with a surprisingly simple conclusion: “nothing—*nothing*—has more impact in the life of a child than positive relationships” (p. 13). Longitudinal findings from the St. Louis Park study strengthen the significance of this conclusion by providing the evidence that most of the assets which show decline are external assets or developmental relationships (Scales et al., 2017).

### Developmental Assets and Psychological Outcomes

Having lineage with the ecological and developmental systems theories (e.g., Bronfenbrenner and Morris, 1998; Lerner et al., 2001), the Developmental Assets Framework and PYD perspective assume a dynamic, bidirectional relationship between the individual and the environment. This bidirectional interaction sets the base for change over time, which is the very essence of ‘development’ (Scales, 2011).

Extensive cross-sectional and longitudinal research conducted primarily in the United States as well as across different cultures have indicated that Developmental Assets are associated with a variety of psychological and academic outcomes. A review of research from 31 countries has indicated that developmental assets are associated with workforce development (e.g., safe and productive employment, job certifications, budgeting skills), educational attainment, health promotion (e.g., hygiene, health-related knowledge), violence mitigation (e.g., being neither a perpetrator or a victim, low normative acceptance of violence), civic engagement, and psychosocial development (e.g., self-efficacy, positive emotionality, interpersonal problem solving skills, leadership) (Scales et al., 2017). In addition, Developmental Assets have been shown to have combinatory effect on thriving outcomes such that the higher the positive developmental resources young people enjoy in their respective environments, the higher the extent of thriving outcomes. Such thriving outcomes include school success, physical health, delay of gratification, overcoming adversity, leadership, value diversity, and prosocial behavior (Scales et al., 2000). Developmental assets buffer against ill-being and strengthen resilience even in the most challenging environments. A study with youth who were exposed to violence showed that positive relationships contributed to the development of resilience over years (Jain et al., 2012).

More recent studies conducted with diverse groups continued to support the contribution of developmental assets to positive outcomes. In a sample of Malaysian college students, external assets (school, friend, family, and community engagement) were found to be linked with prosocial behavior through the mediation of individual developmental assets (i.e., purpose in life and cognitive autonomy) (Kaur et al., 2019). A study with participants from Ghana, Kenya and South Africa indicated that higher levels of developmental assets were linked with better academic performance (Adams et al., 2019). A longitudinal study with American Native Indian early adolescents indicated that developmental assets (i.e., family support, commitment to school, community and peers and personal assets (i.e., self-control) served as protective factors against risky sexual behavior (e.g., sexual debut, the number of sexual partners and condom use frequency) (Greene et al., 2018). In a sample of Portuguese adolescents, a positive association was found between the number of developmental assets and the level of life satisfaction, the internal assets (particularly self-esteem in the positive identity category) having stronger association with life satisfaction than the external assets (Soares et al., 2019). Overall, abundant research showed that both internal and external assets are associated with positive youth outcomes.

### Emerging Adulthood and Developmental Assets

Extensive research validated the utility of developmental assets in positive developmental outcomes in children and adolescents. There is also some but limited evidence showing that developmental assets also play a positive role among older age groups (i.e., college students and emerging adults [EA]). Research showed that higher levels of assets were associated with higher academic success, higher thriving indicators and life satisfaction, and lower risk indicators and pathology (Pashak et al., 2014, 2018). Higher levels of both external and internal assets were found to be associated with healthy pursuit of identity among Turkish EA (Dost-Gözkan and Wiium, 2021).

The most important characteristic of EA is self-discovery and identity seeking in domains of education, professional life, romantic relationships, and worldview (Arnett, 2000). In addition to identity seeking and experimentation, the experience of EA period has also been characterized by indecision/uncertainty, being self-focused, feeling in between and having various life-choices (Arnett, 2000). Growing up, which was a more standardized and predictable project in the past has become an individual project which is less standardized, and which requires individuals to assume more responsibility (Zupan, 2016). Some scholars also stressed that navigating through various alternatives and making meaningful commitments to social roles has become more challenging today because there are more options to choose as compared to previous generations (Ryan and Deci, 2017).

However, despite their challenges, transition periods may provide opportunities to shift from negative to positive pathways as well (Scales et al., 2016). As validated by limited research on EA, PYD and Developmental Assets perspectives may serve useful frameworks that can be utilized to buffer against the challenges of EA, and to facilitate transition from adolescence to adulthood (Pashak et al., 2018). In this respect, focusing on thriving and identifying the factors that can facilitate taking on adult roles is crucial for both research and application.

### A Focus on Norway, Romania, Slovenia, and Turkey

The present research focused on EA from four countries in Europe: Norway, Romania, Slovenia and Turkey, which have both similarities and differences in their social and cultural structures as well as in indices of development in various domains. Norway, Romania, Slovenia and Turkey are geographically located in Northern, Central-Eastern, Southern-Central and South-Eastern Europe, respectively. According to The World Bank (2020), all countries are considered as high-income economies, except Turkey, which is classified as an upper-middle income country. In the 2019 Human Development Index, although all four countries were in the ‘very high development’ category among 189 nations, they differed in their rankings, Norway being the first, Romania the 52nd, Slovenia the 24th and Turkey being the 59th (UNDP, 2019). In the latest World Happiness Report, Norway is the fifth and has shared the top positions with other Nordic countries in the seven versions of the World Happiness Reports; Romania the 47th, Slovenia scores the 33rd, and Turkey the 93rd among 153 nations (Helliwell et al., 2020).

With respect to cultural values defined in Hofstede’s six dimension model (2011), Norway scores high on individualism which reflects a culture where the self is defined in terms of the individual rather than the groups to which the person belongs. Romania, Slovenia, and Turkey, on the other hand, score low in individualism. Low score on individualism indicates a collectivist culture in which the self is defined in terms of group membership. In a culture of collectivism, close and committed relationships are encouraged (e.g., family) (Hofstede Insights, 2020). Individualism-collectivism dimension has implications for child and youth development such that autonomy is given priority in individualist countries whereas in more collectivist cultures it is relatively backgrounded and family ties and intergenerational relations are more foregrounded (Kagitcibasi, 2013). The extent individual members of a society are seen as autonomous beings or as members of the basic unit of family has also implications for family and youth policies countries implement (Yurttagüler, 2016).

Young people constitute valuable assets for their respective countries. In 2019, young people (ages 15–29) constitute 20% out of 5 million in Norway, 16% out of 19 million in Romania, 16% out of 2 million population in Slovenia, and 24% out of 82 million in Turkey (Eurostat, 2019). Education Index (the average of percentages of adult education and the expected year of schooling of children) of Norway, Romania, Slovenia, and Turkey is 0.91, 0.76, 0.89, and 0.71, respectively. International Labour Organization (International Labour Organization [ILO], 2019) reports that the percentage of young people who are neither in employment nor in education (NEET) is 4.8, 6.6, 14.5, and 24.4%, respectively for Norway, Romania, Slovenia, and Turkey among youth between ages 15 and 24. In the latest report of Youth Development Index, which provides both global and specific scores in the areas of health and well-being, education, employment and opportunities, civic engagement and political engagement, Norway, Romania, Slovenia, and Turkey rank the 69th, 35th, 12th, and 90nd, respectively among 183 countries (Commonwealth Secretariat, 2016). Such global statistics highlight that there is variation across the countries with respect to the resources that is necessary for youth people to thrive.

As for the youth policies, all four countries have comprehensive national youth policies. The Norwegian Government’s 2015 Plan on child and youth policy (which covers ages between 0 and 24) sets the following goals: (1) Safe upbringing within the family and local community, (2) Equal rights and opportunities, (3) Participation and influence, (4) High quality service for everyone. The implementation of the youth plan and activities is carried out by local municipalities, which are highly autonomous in their practice. Organizations across different levels of the Norwegian society also provide considerable support for youth activities (Kramer, 2020).

In Romania, different official documents define youth as either between 14 and 35 or 15 and 25. Youth programs, which were initiated in 2001, has mainly targeted facilitation of access to education, transition from education to adulthood, and balancing work and personal life (through childcare and social services). The new National Strategy on Youth, adopted in 2015, has four pillars: (1) Culture and non-formal education; (2) Health, sports and leisure; (3) Participation and volunteering; and (4) Employment and entrepreneurship. All of these pillars are guided by the principle of social inclusion which is considered as a fifth horizontal pillar. The Ministry of Youth and Sports is the body mainly responsible for implementation, coordination and evaluation of the Strategy (Mitulescu and Şerban, 2017).

In the Slovenian national youth strategy, the age range of youth comprises ages between 15 and 29. Slovenian government’s national strategy, adopted in 2006, mainly aims to provide better support for families, to ensure good quality life to youth, to reduce poverty and social exclusion, to ensure adequate welfare benefits to vulnerable youth, to reduce school drop-outs, and to provide vocational and education programs to the unemployed youth. The National Youth Strategy, adopted in 2013, aims to foster intergenerational cooperation and greater solidarity between generations, it also focuses on improving health and welfare of young people, and providing young people a safe start with their careers. The implementation of youth services is being transferred from the public to the private sector; yet non-governmental organizations are also active service providers (Zupan, 2016).

In the Turkish youth policy, individuals between the ages of 14 and 29 are considered as the target group of policy. The governance of the policy is centralized and run by The Ministry of Youth and Sports. The policy paper outlines policy areas, such as education, family, humanitarian values, employment, social inclusion, health and environment, participation and civic consciousness, culture and art, science and technology, intercultural dialog, leisure activities, youth information, voluntary work, and sports (Sener, 2017). Like the Slovenian youth policy, Turkish youth policy has an explicit emphasis on family, where family is deemed as the main unit of the society responsible for the development of children and youth (Yurttagüler, 2016). Turkey is not an EU member, but the youth policy agrees with the EU Youth Strategy (Sener, 2017).

### The Present Study

The Developmental Assets framework stresses that strengths in relationships are crucial for young people regardless of their cultural and socioeconomic background and demographic characteristics, such as sex, sexual identity, ethnicity and race (Scales et al., 2017). In that respect there is the expectation that developmental assets would serve as a common ground that can contribute to positive outcomes and thriving indicators across contexts.

Adopting the PYD and Developmental Assets Frameworks, the present research examined the extent to which external developmental assets are experienced and how these assets are associated with positive identity among emerging adults (EA) in four countries in Europe: Norway, Romania, Slovenia, and Turkey. The present research aimed to contribute to PYD research by focusing on these four countries which have different social, economic, and cultural backgrounds. In addition, we focus on emerging adults on which there is limited research in the field of PYD. In view of the theoretical framework and empirical findings, we expect that the experience of the external assets would contribute to positive identity, although the extent of developmental assets may differ across the four countries.

In the Developmental Assets Framework, the conceptualization of positive identity covers perceived control over one’s future and life (i.e., autonomy and agency) and having good feelings about the self and one’s future (i.e., optimism and hope). In the model proposed by Search Institute and Social Development Research Group, a bidirectional relationship between developmental relationships (social and family relationships) and developmental outcomes (e.g., health, ethical behavior, life skills, educational and occupational commitment and engagement, and civic engagement) is proposed to be mediated by developmental processes (identity, agency, and commitment to the community) (Scales et al., 2016). Indeed, a recent study from Turkey has shown that the internal asset of positive identity mediated the relationship between developmental assets and identity statuses and identity dimensions in two groups of EA, respectively (Dost-Gözkan and Wiium, 2021). Based on the Scales et al. (2016) theoretical model and empirical evidence that positive identity can be a catalyser for thriving, the present study focused on the relationship between external assets and positive identity.

One of the external assets, the constructive use of time, was not included in the present study since it consistently had very low level of internal consistency in earlier studies. Constructive use of time, in fact, was intentionally developed to include multidimensional experiences, which psychometrically renders the scale susceptible to low internal consistency (Scales, 2011). As expected, constructive use of time was found to be psychometrically the least reliable as well as the least consistently experienced asset category across 31 countries (Scales et al., 2017). Therefore, the present research does not include constructive use of time.

The present study investigates two questions:

(1) Are there any country differences in the extent to which external assets of support, empowerment, and boundaries and expectations and the internal asset of positive identity are experienced?

(2) How are external assets associated with positive identity across the four countries?

We hypothesized that each of the external assets would be positively linked with the positive identity across the four countries, although there might be differences in the experience of assets. Depending on the relative salience of assets in the four countries, the magnitude of the association between the external assets and positive identity may show variation across countries.

## Materials and Methods

### Participants

As shown in Table 1, a total of 2055 young adults (Norway = 488, Romania = 255, Slovenia = 561, Turkey = 751) ranging in age between 18 and 28 (*M*_*age*_ = 20.41; *SD* = 2.04) participated in the present study. Country-wise statistics can be seen in Table 1. ANOVA analysis with Bonferroni adjusted post-hoc tests indicated that there were age differences across the four samples, *F*(3, 2053) = 28.22. Slovenian participants were slightly older than Norwegian, Romanian and Turkish participants whose ages were similar.

**TABLE 1:** Study variables across four European countries: Norway, Romania, Slovenia, and Turkey.

	Norway	Romania	Slovenia	Turkey	Total
Study variable	*n* = 488	*n* = 255	*n* = 561	*n* = 751	*n* = 2055
Mean age (SD)	20.13 (1.38)	20.29 (1.91)	21.07 (2.57)	20.15 (1.89)	20.41 (2.04)
**Gender %**					
Male	27.0	57.9	21.5	29.8	30.4
Female	73.0	42.1	78.5	70.2	69.6
**Mother’s education %**					
Vocational, technical, polytechnic or university Secondary school or lower	82.6 17.4	50.8 49.2	47.6 52.4	35.3 64.7	51.7 48.3
**Father’s education %**					
Vocational, technical, polytechnic or university Secondary school or lower	78.7 21.3	51.6 48.4	37.6 62.4	43.8 56.3	51.3 48.7
**Cronbach’s alpha of assets and outcome**					
Support	0.81	0.65	0.75	0.81	0.77
Empowerment	0.74	0.71	0.72	0.72	0.73
Boundaries and expectations	0.78	0.68	0.71	0.81	0.75
Positive identity	0.88	0.83	0.84	0.88	0.86

Majority of the participants were females (69.6%). Chi-square analyses showed that there were sex differences across the countries. While females were represented at higher percentages in Norwegian, Slovenian, and Turkish data, in the Romanian data the sex distribution was slightly in favor of males, χ*^2^*(3, 2046) = 114.427, *p* < 0.001. Parental demographics showed that about half of the mothers (51.7%) and fathers (51.3%) had a vocational school or college degree, and 48.3% of mothers and 48.7% of fathers had a secondary or lower school degree. Chi-square analysis indicated that the percentage of mothers with higher education was much higher than the percentage of mothers with lower education in the Norwegian sample, while it was the reverse in the Turkish sample. In the Slovenian and Romanian samples, the percentages the two education groups were similar, χ*^2^*(3, 2035) = 267.87, *p* < 0.001. Similarly, the percentage of fathers with higher education was higher than the percentage of fathers with lower education in the Norwegian sample, while it was the reverse in the Turkish and the Slovenian samples; the percentages of the two educational categories for fathers were close to each other in the Romanian sample, χ*^2^*(3, 1992) = 197.88, *p* < 0.001.

### Data Collection Procedure

Data were collected from the college students using online platforms, such as SurveyXact (in Norway), Qualtrics (in Turkey), and paper and pencil (in Romania). Before the data collection, participants were informed about the goal and procedures of the study. At the beginning of the questionnaire informed consent was obtained from the participants. The study in Norway was approved by the Regional Committees for Medical and Health Research Ethics. In Romania, data collection was undertaken in educational settings, mainly from undergraduate and postgraduate university students located in central part of the country. Students received no extra credit for their participation. In Turkey, The Ethical Board of Ozyegin University endorsed the study. In Slovenia, the present study as part of a larger study investigating longitudinal pathways for positive youth development in the context of migration was approved by the Slovenian Research Agency. After obtaining informed consent, the students responded either online or on paper. The time was not limited.

The data was collected with multimode methodology using computer-based and paper and pencil methodologies. Mixing data collection modes is an option depending on the convenience of access to participants, avoidance of incomplete data and (un)availability of computerized equipment for data collection (de Leeuw, 2005). Yet there are also concerns for the equvalence of the data collected with different modes. Although responses of the participants may be affected by the mode of data collection, favoring the computer-based surveys in accuracy, research shows that respondents’ answers differ only in personally sensitive topics but not in mundane topics. Research also shows that findings differ with respect to the mode of data-collection in cases where the responses of the participants are not anonymous in paper-pencil surveys. When anonymity was assured there is no difference in the responses of the groups which differ in the mode of the survey regardless of the personal sensitivity of the question (Knapp and Kirk, 2003). In our sample, anonymity as well as the confidentiality of anonymous data was assured. Also our questions were not personally sensitive (e.g., about sexual and criminal behavior) as reported by previous studies. Therefore, we confidently used the dataset collected with multimode method.

### Measures

#### Demographics

Participants reported their age and their sex (i.e., male or female) as well as the educational level of their mother and father, as a proxy for socioeconomic status (SES) (see Table 1 for details).

#### Developmental Assets Profile

The original questionnaire was developed by Benson (1990, 2007) consisting of 40 assets. Search Institute (2007) developed Developmental Assets Profile consisting of 58 items that reflect internal and external assets that address multiple contexts (e.g., family and school). Internal asset category includes four assets (*commitment to learning*, *positive values*, *social competencies* and *positive identity*), and external assets include four assets (i.e., *support*, *empowerment*, *boundaries and expectations* and *constructive use of time*). Participants are asked to rate the extent to which they experienced each asset on a 4-point scale (1-not at all or rarely, 2- somewhat or sometimes, 3- very or often to 4-extremely or almost always).

In the present study, the three external assets (*support, empowerment*, *boundaries and expectations*) and one internal asset (*positive identity*) were used. As per the present research concerns, external assets (except constructive use of time) were considered as independent variables, and positive identity was framed as the dependent variable. Sample items were: “I ask my parents for advice” for support (7 items), “I am given useful roles and responsibilities” for empowerment (6 items), “I have a family that gives me with clear rules” for boundaries and expectations (9 items), “I feel that my life has a purpose” for positive identity (4 items).

The Developmental Assets Profile was originally developed in English and it was translated in the formal local language of the respective countries for data collection. In Norway, *Amesto Translations*, a Scandinavian company that offers translation and interpretation services, translated the questionnaire. In Romania, two researchers proficient in English and Romanian were involved in the translation, back-translation and pre-testing of the questionnaire. In Turkey, three Turkish-English bilingual researchers conducted the translation, back-translation and questionnaire validation. In Slovenia the translation and back translation was conducted by two researchers, both fluent in English.

Cronbach’s alphas of the assets, ranging from 0.65 to 0.88 (Table 1), are in the range of acceptable to very good and are comparable to the reports given in previous studies (Scales et al., 2000; Scales, 2011).

### Data Analysis

About 12% of the participants had missing on 6 or less items, where 11% had missing on one or two items, while the remaining 1% had missing on between 3 and 6 items. Pairwise deletion was used to handle missing cases in descriptive analysis. Descriptive analyses and reliability tests were undertaken to assess the distribution of the demographic variables and the internal consistencies of items measuring the assets on *support*, *empowerment*, *boundaries* and *expectations*, as well as *positive identity*. Composite scores reflecting the number of assets reported for each asset category were created after recoding the 4-point Likert scale into a binary one, where response alternatives 1 and 2 were recoded as asset not present, and 3 and 4 recoded as asset present.

Country similarities and differences in developmental assets were examined performing multivariate univariate analyses of covariance controlling for the participants’ sex, age, maternal and paternal education levels, which were associated with study variables and showed variation across countries.

Prior to the assessment of the association of *support*, *empowerment*, and *boundaries* and *expectations* with *positive identity*, measurement invariance (i.e., configural invariance, metric invariance and scalar invariance) was examined across the four countries (i.e., Norway, Romania, Slovenia and Turkey) by conducting a set of Multigroup Confirmatory Factor Analyses (MGCFA) on the items measuring the asset categories, with Mplus (Muthén and Muthén, 1998-2017). Configural invariance is when the asset items load onto the latent factor (e.g., support) in the same fashion across countries, while for metric invariance, the factor loadings of the asset items measuring the asset categories are the same across countries. For scalar invariance, it is not only the factor loadings of the asset items that are identical but the intercepts as well. Thus, measurement invariance was conducted to be able to make meaningful comparisons across countries (see Spini, 2003; Putnick and Bornstein, 2016).

Following measurement invariance, three path analyses: (1) a constrained model, (2) an unconstrained model, and (3) a trimmed model (where a previously unconstrained path was constrained) were conducted to ascertain the best-fit model. Gender and age along with father and mother’s educational background were treated as covariates and constrained in all three models. To determine the best-fit model, chi-square tests and fit indices, such as the Tucker Lewis Index (TLI; acceptable > 0.90), the Root Mean Square Error of Approximation (RMSEA; acceptable below 0.08), and Comparative Fit Index (CFI; acceptable above 0.90) (Hu and Bentler, 1999; Brown, 2015) were used.

## Results

### Measurement Invariance Across Countries

In a series of Multigroup Confirmatory Factor Analyses (MGCFA) conducted for the asset variables and positive identity to assess measurement invariance (configural, metric and scalar), full configural and metric invariance were established for all variables, and full scalar invariance was reached for *positive identity*. Partial scalar invariance across countries was established for *support*, *empowerment* and *boundaries and expectations*, where equality across countries had to be relaxed for the intercept of several items measuring these asset variables (Table 2). Researchers acknowledge that despite being an ideal criterion, full measurement invariance is not quite possible across all measurement testing steps (i.e., configural, metric, scalar), and it is becoming a common practice to allow partial variation in certain circumstances (for a detailed discussion see, Guenole and Brown, 2014; Putnick and Bornstein, 2016). It has been shown that ignoring only one noninvariant factor loading (i.e., metric noninvariance) or intercept (i.e., scalar noninvariance) does not yield significant bias in parameter estimation; however, ignoring full noninvariance of an item (e.g., metric and scalar noninvariance) yields significant bias. In our sample, none of the items were fully noninvariant across the measurement testing steps. Therefore, considering that full configural and metric invariance were achieved for all variables in the present study, full invariance was achieved for positive identity and partial invariance was achieved for external assets, we proceeded with an approach which allows for partial invariance (Guenole and Brown, 2014; Putnick and Bornstein, 2016).

**TABLE 2:** Measurement invariance models for developmental assets by country.

Model	Model fit indices			
	χ2 (df)	RMSEA	90% CI RMSEA	CFI/TLI
**Support**				
Configural invariance	110.395 (32)	0.069	0.055–0.083	0.966/0.911
Metric invariance	157.261 (50)	0.065	0.053–0.076	0.954/0.922
Scalar invariance	425.559 (68)	0.101	0.092–0.110	0.845/0.809
Partial scalar invariance	177.630 (55)	0.066	0.055–0.077	0.947/0.919
**Empowerment**				
Configural invariance	94.612 (24)	0.076	0.060–0.092	0.961/0.901
Metric invariance	133.707 (39)	0.069	0.056–0.082	0.947/0.919
Scalar invariance	259.271 (54)	0.086	0.076–0.097	0.885/0.873
Partial scalar invariance	167.391 (48)	0.070	0.058–0.081	0.933/0.917
**Boundaries and Expectations**				
Configural invariance	150.397 (56)	0.057	0.046–0.068	0.965/0.911
Metric invariance	206.873 (80)	0.056	0.046–0.065	0.953/0.914
Scalar invariance	706.768 (104)	0.106	0.099–0.114	0.779/0.694
Partial scalar invariance	240.245(94)	0.055	0.046–0.064	0.946/0.918
**Positive Identity**				
Configural invariance	20.258 (4)	0.089	0.053–0.129	0.992/0.953
Metric invariance	43.127 (13)	0.067	0.046–0.090	0.985/0.973
Scalar invariance	84.772 (22)	0.075	0.058–0.092	0.970/0.965

*χ^2^ = Chi-Square; df = degrees of freedom; CFI = Comparative Fit Index; TLI = Tucker Lewis Index; RMSEA = Root Mean Square Error of Approximation; CI = Confidence Interval; Configural – equivalence of model form; Metric – equivalence of factor loadings; Scalar – equivalence of item intercepts or thresholds.*

### Descriptive and Correlation Analyses

In results presented in Table 3, information on the mean scores indicates that the number of assets reported by participants was above average for all three external asset variables and positive identity. Moderate correlations were observed among the asset variables and positive identity, ranging from 0.33 to 0.43, with the highest correlation registered between empowerment and positive identity (*r* = 0.43, *p* < 0.01). In addition, weak correlations ranging from −0.00 and 0.11 were found between the demographic variables (age, gender, and parents’ education) and the asset variables together with positive identity (Table 3).

**TABLE 3:** Descriptive statistics and correlations among study variables for the total sample.

Study Variables	1	2	3	4	5	6	7	8
(1) Age	–	0.03	0.03	0.05*	−0.07**	0.06**	−0.04	−0.00
(2) Gender (1 = Male; 2 = Female)		–	0.04	0.03	0.11**	0.10**	0.10**	−0.06**
(3) Mother’s education			–	0.52**	−0.02	0.04	0.04	−0.04
(4) Father’s education				–	−0.02	0.02	0.05*	−0.05*
(5) Support					–	0.53**	0.59**	0.37**
(6) Empowerment						–	0.50**	0.43**
(7) Boundaries and expectations							–	0.33**
(8) Positive identity								–
*Mean (SD)*	20.41 (2.04)	– –	1.52 (0.50)	1.51 (0.50)	4.82 (1.74)	5.04 (1.28)	6.38 (1.97)	2.96 (1.38)
*Range*	18–28	1–2	1–2	1–2	1–7	1–6	1–9	1–4

SD = Standard Deviation; *p < 0.05; **p < 0.01.

### MANCOVA and ANCOVAs Examining Country Similarities and Differences in Developmental Assets

A MANCOVA was performed to see if there were any country differences in developmental assets examined in the present study. The multivariate effect was significant, Pillai’s Trace = 0.08, *F*(5898, 12.000) = 13.263, *p* < 0.001, partial η^2^ = 0.026. As shown in Table 4, all univariate effects were significant, indicating that there was a significant country difference in each of the developmental assets. Bonferroni adjusted pairwise comparisons indicated that EA in Slovenia had the highest level of overall external assets. EA in Slovenia also reported more support assets, which was not significantly different from the reports of EA in Turkey, but was significantly higher than the reports of EA in Norway and Romania. EA in Turkey also reported significantly higher experience of support compared to EA in Norway and Romania. The support scores of Norwegian and Romanian EA were similar. In order to understand from which items, the country differences were coming, the frequencies of participants who endorsed that they experience a specific asset was also examined at the item level. As can be seen in Table 5, the difference in the experience of support was related to items measuring support that was received from other adults; the experience of support from family was similar across the four countries.

**TABLE 4:** Univariate statistics for the cultural comparisons of support, empowerment, boundaries and expectations, and positive identity.

Dependent Variable	All groups (n = 1975)	Norway (n = 463)	Romania (n = 250)	Slovenia (n = 532)	Turkey (n = 730)	F (3, 1974)	Partial η^2^	
Support	4.80 (0.41)	4.51 (0.08)	4.60 (0.11)	5.16 (0.08)	4.93 (0.06)	12.50***	0.019	S > N, R*** T > N**, R*
Empowerment	5.01 (0.03)	4.95 (0.06)	4.76 (0.08)	5.45 (0.06)	4.94 (0.05)	23.64***	0.035	S > N, R, T***
Boundaries and Expectations	6.38 (0.05)	6.48 (0.10)	6.09 (0.13)	6.73 (0.09)	6.22 (0.07)	8.86***	0.013	S > R, T***
Positive Identity	2.98 (0.03)	2.73 (0.07)	3.10 (0.09)	3.00 (0.06)	3.10 (0.05)	6.51***	0.010	R > N** S > R* T > N***

N = Norway, R = Romania, S = Slovenia, T = Turkey Gender, age, and maternal and paternal education levels were controlled for in the analyses. Composite scores reflecting the number of assets reported for each asset category were created after recoding the 4-point Likert scale into a binary one, where response alternatives 1 and 2 were recoded as asset not present, and 3 and 4 recoded as asset present. The highest score for Support, Empowerment, Boundaries and Expectations, and Positive Identity can be 7, 6, 9, and 4, respectively. p*** < 0.000, p** < 0.01, p*< 0.05

**TABLE 5:** Percentage of emerging adults who experienced external asset and positive identity per item.

	Norway	Romania	Slovenia	Turkey	

Support	%	%	%	%	χ*^2^*
Support from the family	92.62	92.16	95.19	92.14	*ns*
Asking parents advice	72.75	73.52	72.86	70.59	*ns*
Support from other adults	50.31	41.90	**79.50**	**73.97**	185.23***
Good and caring neighbors	30.77	30.83	**40.46**	33.24	13.34***
Caring and encouraging departmental environment	63.77	50.59	**73.98**	**74.33**	62.74***
Parental help to succeed in school	72.69	**87.75**	61.85	73.70	60.88***
Parents good at talking about things	76.025	77.08	**82.53**	75.73	10.10*
**Empowerment**					
Feeling valued and appreciated	82.34	66.27	**88.59**	77.23	61.56***
Having useful roles and responsibilities	72.99	69.17	**85.00**	75.63	33.89***
Included in family tasks and decisions	77.66	76.86	81.82	80.77	*ns*
Feeling safe at the university	83.51	81.57	**91.98**	87.60	24.54***
Having a safe neighborhood	88.09	83.53	**96.26**	77.57	95.79***
Feeling safe at home	95.07	95.62	96.97	94.40	*ns*
**Boundaries and Expectations**					
Family knowing whereabouts	83.78	82.68	**89.13**	80.35	18.57***
Department with clear rules	80.08	79.92	**87.34**	82.24	12.30**
Neighbors watching out for the young	17.08	35.04	28.34	**66.93**	343.21***
Adults who are good models	**87.06**	67.84	62.03	72.99	85.85***
Friends setting good examples	**85.60**	70.59	**87.50**	69.07	89.39***
Professors encouraging development	63.45	68.90	**71.43**	66.04	8.44*
Family with clear rules	**70.23**	43.14	**75.71**	36.58	260.15***
Department enforcing rules fairly	**80.25**	68.63	72.45	64.39	36.98***
Parents encouraging school success	**92.55**	83.40	**91.76**	85.56	26.64***
**Positive Identity**					
Control over one’s life and future	**54.62**	71.26	74.33	78.43	86.18***
Feeling good about self	73.36	84.31	73.26	76.07	13.60**
Life with a purpose	**63.93**	80.39	74.29	76.33	32.25***
Feeling good about one’s future	78.40	78.82	74.29	77.97	*ns*

Values in bold are the highest percentages for each item across the four countries. *p < 0.05, **p < 0.01 and ***p < 0.000.

The experience of empowerment was also the highest among EA in Slovenia and it was significantly higher than the respective reports of EA in the other three countries (which were similar). The examination of item percentages indicated that the percentage of Slovenian EA was notably higher on items measuring feelings of being valued and appreciated by others, having useful roles and responsibilities, and feeling of security at school and neighborhood (Tables 4, 5).

The reports of boundaries and expectations were also the highest among Slovenian EA, though it was not significantly different from the Norwegian EA. Norwegian, Turkish and Romanian EA were similar in their experiences of boundaries and expectations. Differences between Slovenian, Romanian and Turkish EA were on items related to rules enforced by the family and school and having exemplar friends. Finally, positive identity scores of EA from Norway were lower than EA from Romania, Slovenia, and Turkey. The differences were stemming from items pertaining to sense of control over one’s life and future, and the feeling that one’s life has a purpose (Tables 4, 5).

### Path Analysis of the Asset Variables and Positive Identity

Having established (partial) scalar invariance for the asset variables and *positive identity*, three path analyses assessing the associations between the asset variables and *positive identity* were conducted. The unconstrained model, (χ*^2^*(12, 1977) = 44.635, *p* < 0.001, *CFI* = 0.961, *RMSEA* = 0.074), turned out to be significantly better than the constrained model (χ*^2^*(21, 1977) = 75.701, *p* < 0.001, *CFI* = 0.935, *RMSEA* = 0.073). In a subsequent series of path analysis, the paths between the three asset variables (*support, empowerment* and *boundaries and expectations*) and *positive identity* were assessed one at a time to determine the path that differed significantly across countries. *Support* did not, and thus in a trimmed path analysis, the path between *support* and *positive identity* was constrained. The resulting fit indices were χ*^2^*(15, 1977) = 54.516, *p* < 0.001, *CFI* = 0.953, *RMSEA* = 0.073. Results of the trimmed path analysis indicated that *support* was weakly but significantly associated with *positive identity* (*B* = 0.09, *p* < 0.01) in all four countries. *Empowerment* had significant association with *positive identity* for the four countries (ranging from 0.18 to 0.41), although the association was moderate for Norway and Slovenia, and weak for Romania and Turkey (Table 6). Finally, a weak but significant association (ranging from 0.09 to 0.11) was observed between *boundaries and expectations* and *positive identity* for all countries except Romania, which showed no significant association between the two variables.

**TABLE 6:** Positive identity by external developmental assets.

*Predictors*	*Constrained model*	*Unconstrained model*^†^					*Trimmed model*^‡^				
Support	0.10**		0.05 _N_	0.14** _R_	−0.02 _S_	0.14** _T_	0.09**				
Empowerment	0.31**		41**	0.17**	0.47**	0.25**		0.40** _N_	0.18** _R_	0.41** _*S*_	0.28** _T_
Boundaries and expectations	0.08**		0.11**	0.03	0.16**	0.07**		0.10**	0.04	0.11**	0.09**
Age	0.00	0.00					0.00				
Gender	0.04	0.06					0.07 (*p* = 0.051)				
Mother’s education	0.28**	0.25**					0.25**				
Father’s education	−0.27**	−0.27**					−0.27**				
Model chi-square	χ^2^ = 75.701 df = 21 *p* < 0.001	χ^2^ = 44.635 df = 12 *p* < 0.001					χ^2^ = 54.516 df = 15 *p* < 0.001				
Fit indices	CFI = 0.935 RMSEA = 0.073	CFI = 0.961 RMSEA = 0.074					CFI = 0.953 RMSEA = 0.073				

Unstandardized coefficients from Mplus Path analyses. ^†^Unconstrained for the assets; ^‡^Unconstrained for only empowerment and boundaries and expectations as unconstrained path for support did not produce significant Chi square difference; Chi square difference between constrained and unconstrained models is significant: χ^2^ = 31.066, df = 9, *p* < 0.01; **p < 0.01; N – Norway; R – Romania; S – Slovenia; T - Turkey.

## Discussion

The present study examined the extent to which external assets (support, empowerment, and boundaries and expectations) were experienced among emerging adults (EA) and how these assets were linked to the level of positive sense of self in four European countries, Norway, Romania, Slovenia, and Turkey. These four countries have both similarities and differences in their cultural organization of family relationships (e.g., individualism-collectivism), and in the indices of development in various domains (e.g., educational, youth, and economic development). Except Norway (standing high on individualism), all countries are classified as collectivist countries (Hofstede Insights, 2020). Although differing in ranking, all four countries are under the category of ‘very high development’ in Human Development Index (UNDP, 2019). Except Turkey (an upper-middle class country), all countries are high-income countries (The World Bank, 2020). When these four countries are compared with each other in their standing in the development indices such as human development index, education index, youth development index, the percentage of people neither in education nor in employment or training (NEET), and happiness index, Norway has highest development ranking, and Romania and Slovenia stand between Norway and Turkey which has the lowest ranking (Commonwealth Secretariat, 2016; UNDP, 2019; Helliwell et al., 2020). All four countries have comprehensive youth policies in line with the European Youth Strategy.

The results of the present study indicated both similarities and differences in EA’s experiences of external developmental assets. As expected, despite differences in the experience of assets, all external assets were positively linked with positive identity across the four countries (except for boundaries and expectations, which was not a significant predictor of positive identity in Romanian data). The higher the level of support EA perceived from the family, school, and other adults, the higher was their positive sense of identity. Likewise, the higher the empowerment (i.e., feeling valued and appreciated by others, being given responsibilities and roles, having a sense of security and safety in one’s social environment), the higher was the sense of positive identity. Finally, the higher the boundaries and expectations (i.e., structure provided in the form of clear rules, boundaries, and standards of achievement, having adult role models, and having friends setting good examples) the higher was positive identity.

The finding that external assets are associated with positive identity is in line with the extant literature showing positive associations among the assets, and between the assets and positive outcomes, such as academic success psychological wellbeing (Jain et al., 2012; Scales et al., 2017; Greene et al., 2018; Adams et al., 2019; Kaur et al., 2019; Soares et al., 2019). In view of this literature, the positive association between external assets and positive identity was expected. The novelty of the present findings lies in the fact that the significant association between external assets and positive identity holds across the four countries despite differences in the extent of external assets EA experience in their social environments. In the present study, the outcome variable was positive identity which is one of the internal assets framed in the Developmental Assets Framework. Positive identity assesses perceived control over one’s future and life, and positive feelings about one’s self and future. In other words, positive sense of identity refers to a sense of agency, self-determination, optimism and hope for one’s life and future. In a model Scales and his colleagues (2016) proposed for successful transition to adulthood, healthy developmental relationships are linked with foundational developmental processes such as identity (autonomy), agency (competence) and commitment to community (relatedness), which in turn are linked with developmental outcomes. The model proposes that developmental processes (which comprise the basic psychological needs proposed by the self-determination theory-SDT) function as mediators between the assets and developmental outcomes (Scales et al., 2016).

The present study validates the links between external assets and positive sense of identity which encompasses the three basic psychological needs. As Scales et al.’s (2016) model proposes, basic psychological needs theory of SDT (Ryan and Deci, 2017) frames a compelling perspective which can explain how and why external assets are linked with positive identity. SDT states that autonomy (being the driver of one’s actions and acting based on one’s volution), relatedness (having supportive relationships), and competence (feeling effective in one’s interaction with the environment and having a sense of mastery in the tasks engaged) are the three basic needs that need to be gratified for optimal human development and functioning in any social context (Ryan and Deci, 2017). Support experienced in the environment might be especially gratifying the need for autonomy and encouraging the individual to take initiations and responsibilities that will contribute to awareness of one’s motivations and actions and become more self-determined, hence develop a sense of control over one’s life and future. Empowerment which entails that the young being included in family decisions and having useful roles and responsibilities, as well as the sense of safety and security in social environment might be fostering both sense of autonomy and feeling of competence. Finally, boundaries and expectations provide a structure with role models, standards of excellence, and rules set by the school and the family. According to SDT, structure is a very important component of competence-building environments (Ryan and Deci, 2017). Hence boundaries and expectations may be uniquely contributing to positive identity through setting up the necessary environmental conditions that will foster competence.

The model tested in the present study contributes to the literature by confirming that developmental relationships are linked to the positive sense of identity across the four countries which differ in their cultural values, global indices of economic, human, and youth development, and subjective well-being (Commonwealth Secretariat, 2016; UNDP, 2019; Helliwell et al., 2020; Hofstede Insights, 2020; The World Bank, 2020). The findings also provide support to the claim that developmental assets promote thriving regardless of individuals’ cultural background (Scales, 2011).

As for the extent of the experience of external assets and positive identity, the data from the four countries indicated high scores (in the fourth or third quartiles). Nevertheless, there were notable similarities and differences across the countries. One prominent finding was that Slovenian EA had highest level of overall external assets (although the level of support was similar to the reports of the EA from Turkey, and the level of boundaries and expectations is similar to that reported in Norway). A closer examination of the item-level percentages shows that the country-level differences in support was coming from support perceived from other adults and the department (which were higher for Slovenian and Turkish EA), while support from the family was similar and very high across the four cultures. Indeed, country sheet on youth policy in Slovenia indicates that Slovenian youth finds educational environment quite friendly and socially oriented (Zupan, 2016). This is reflected as a strong and salient asset in the present findings.

Slovenian EA also reported to experience significantly more empowerment than EA from the rest of the three countries, the empowerment scores of which were similar. Item level percentages showed that the percentage of Slovenian EA who reported to experience empowerment or having competence-building experiences (i.e., having useful responsibilities and roles and getting competence feedback in the form of appreciation from others) was higher than the respective percentages of EA from the three countries. The feeling of safety and security at home and being included in family decisions were similar across the four countries. Overall, findings reflecting the experience of support and empowerment indicate that family is a significant developmental asset common across the four countries regardless of the cultural characteristics of individualism (i.e., Norway) and collectivism (Slovenia, Romania, and Turkey).

Among the four countries, however, the emphasis on family is quite clear in the national youth policies of Slovenia and Turkey (Turkish Ministry of Youth and Sport, 2012; Zupan, 2016). In the Turkish youth policy, family is explicitly claimed to be the most important social unit which determines the structure of the society and is deemed responsible for providing material and moral support for its members (Yurttagüler, 2016; Açıkgöz et al., 2019). This might reflect an intergenerational relationship pattern already embedded in the culture. One view state that as young people are seen as members of the family rather than as autonomous individuals, the policies aimed to support young people focus on the family (Yurttagüler, 2016). The emphasis on family in policy documents may also reflect a post-welfarist approach in which the state disclaims responsibility and utilizes the family for the provision of social benefits, a policy approach which might have negative ramifications for young people’s transitioning to adulthood (such as prolonged dependence on family and taking adult roles even later) (for a discussion see, Chevalier, 2016; Açıkgöz et al., 2019). Similar concerns were voiced in the Slovenian youth policy country sheet. Although young people are quite content with their family relationships (in fact, having the best relationships with parents among EU-27), and enjoy the inclusiveness and friendliness of the education system, they are concerned about the poor connection between the education system and the demands of the labor market, which is highly unpredictable compared to countries in EU-27 (Zupan, 2016). It is stated that for the Slovenian youth, the condition for transition to adulthood is characterized by a substantial support by the family and prolonged involvement in a friendly education system, and a quite uncertain labor market that young people will endeavor to take part. Youth policy report on Slovenia concludes that the social structure makes it harder to take on adult roles such as steady employment, and starting a family (Zupan, 2016).

The present research also showed that Slovenian and Norwegian EA had higher experience of boundaries and expectations (i.e., having families and schools enforcing clear rules and having good exemplar friends) than EA from Romania and Turkey. Boundaries and expectations category was also the strongest external asset which predicted positive identity for Slovenian and Norwegian EA in the present results. Clear rules, boundaries and expectations provide a helpful structure for the growing child and the young person. Structure has been considered as one of the competence-building characteristics of social environment in the self-determination theory (SDT) (Ryan and Deci, 2017). A closer examination of item level percentages indicates that compared to Romania and Turkey, Slovenian and Norwegian family structure stands out with setting clear rules and with parents encouraging success. Department with clear rules, encouraging professors, and family knowing whereabouts were the discernible items with much higher percentages in Slovenian dataset. This finding is congruent with other studies which reports youth contentment with the educational institutions and family involvement in youth development in Slovenia (Zupan, 2016). The experience of Norwegian EA stands out on items related to department enforcing rules fairly and with adults setting good models. These findings underscore the importance of structure provided in developmental relationships for thriving.

Finally, although reports on positive identity was high across the four countries, Norwegian EA reported lower levels of positive identity compared to EA from the rest of the three countries. Item level differences indicated that relatively fewer Norwegian EA reported to feel control over their future and feeling that their life has a purpose. This finding may look surprising given that Norway ranks among the first countries in global indices of human development, youth development, and economic prosperity. Eurostat data provides additional insights to make sense of this finding (Eurostat, 2020a). Eurostat 2018 life satisfaction data shows that 40.8% of Norwegian young people (ages between 16 and 24) endorse that they are highly satisfied with their lives, 49.6% indicate that they have medium level life satisfaction, and only 9.7% indicate that their life satisfaction is low. While the respective statistics for Slovenia, Romania and Turkey are as follows: high satisfaction: 44.2, 45.7, and 16%; medium satisfaction: 49.2, 47.3, and 42.7%; and low satisfaction: 6.5, 7.8, and 41.3%. When this data was restrained to young people ages between 16 and 24 who have tertiary education, while the percentages of highly satisfied EA increases for Slovenia and Romania to 51.2 and 60.3%, respectively, and stays similarly low for Turkey (14.5%), it shows an opposite trend and drops from 40.8 to 24.8% for Norway.

The percentages in two specific items in the present study allows a meaningful interpretation when considered in view of the statistics of the percentages of youth living with their parents, together with Eurostat life satisfaction statistics. Eurostat 2019 data shows that while in Slovenia, Romania, and Turkey the percentage of young people (ages between 16 and 24) living with their parents is 91.5, 83.9, and 84.5%, respectively, it is 56.6% for Norway (Eurostat, 2020b). The difference becomes even more drastic when percentages in the older age groups are compared. For example, for the age group between 25 and 29, while the percentage of EA living with their parents drops by 32 to 37% for the three countries, it drops by 47.5% and becomes 9% for Norway. These statistics show that there is a notable cultural difference between Norway and the rest of the three countries in the social clock related to the age young people are expected to leave their parents’ home and stand on their feet to start an independent life. This might be the reason why in the present study there is relatively lower percentage of Norwegian EA endorsing items related to control over one’s life and future and having a life with a purpose. The expectations to take on an adult responsibility on such an early age might create some uncertainty and distress that Norwegian young people need to tackle much earlier than EA in Slovenia, Romania, and Turkey.

Previous research has shown that as age increases young people perceive fewer external assets in their respective environment. This was partially confirmed in the present data which shows an inverse relationship between age and support, but a positive relationship between age and empowerment. Scales et al. (2016) interpret the inverse relationship between age and external assets saying that elements of the social context such as family, school, neighborhoods and other organizations that young people partake in fail to prepare young people to transition to adult roles. The present data suggests an alternative explanation to be further investigated in future. It is possible that adult assistance takes the form of empowerment which involves giving more responsibilities and adult role related expectations rather than providing mere support in the form of caring, encouragement, giving advice, talking about things and helping in school achievement (which the present support scale assesses). This may indicate a developmental shift in the relationship between the growing young and adults in helping the young in their transition to adult roles. Future research might consider examining the relative salience and significance of external assets with respect to age.

External assets in the Developmental Assets Framework encompass the immediate social context which includes family, school, peers, and other adults in close proximity. These developmental relationships fit well in the microsystem (family, school, peers, neighborhood) in the Bronfenbrenner’s model (Bronfenbrenner and Morris, 1998). Bronfenbrenner’s ecological model provides a helpful template which elucidates the dynamic and interactive systems involved in the ecology of the development of an individual. In the ecological model, the exosystem, which encompasses legal regulations, social policies and their implementations, the macrosystem, which embeds cultural values and belief systems (e.g., age related expectations, social clock, ethnotheories about the definition and capabilities of a young person and an adult), and the chronosystem (time related changes) also play significant roles in development both directly and indirectly through their influence on the actors and relationships in the microsystem and the macrosystem.

A comparison of data from Turkey in the present study and in other resources (e.g., Eurostat) may provide a notable case that can be discussed in view of the relative role of systems in the ecological model. The present findings show that EA from Turkey is faring well in all assets, either scoring higher from (e.g., support) or similar to (e.g., empowerment, boundaries and expectations, and positive identity) two or three countries. The results indicate that Turkish EA benefit from developmental relationships in their microsystem as much as EA from Norway, Slovenia, and Romania. However, other youth indices pinpoint that there are limitations that Turkish EA have access to resources. For example, the percentage of EA having material and social deprivation is 14.2% in Turkey, while these percentages are 10, 1.4, and 1.3% for Romania, Slovenia, and Norway, respectively (Eurostat, 2020c). EA who are neither employed nor continue training or education also constitute 29.1% of young people with tertiary education in Turkey (being the third highest among EU countries), while the respective percentages are 8.5% for Romania, 7.8% for Slovenia, and 4.1% for Norway (Eurostat, 2020d). When these percentages are evaluated in combination with the findings of the present research, it is clear that although family and developmental relationships in the immediate social environment are crucial assets, the resources they can provide have limits, especially if the material resources and opportunities and the social capital needed are beyond their access.

The limitations of social policies that target improvement of individual resources while not paying enough attention to structural inequalities was discussed in a seminal article by Albee and Ryan-Finn (1993) on social justice and prevention science. Albee and Ryan-Finn (1993) proposes a formula which defines prevalence of mental and emotional distress as a function of poor environmental resources, resulting from lack of social justice, divided by the assets of the individual or the group to combat with such societal inequalities. Fisher et al. (2012) adapted this formula to the field of youth development and defined the incidence of developmental problems as a function of poor access to social capital and material resources divided by the assets of the individual, family, and the broader community. Fisher et al. (2012) further highlighted that the more entrenched the structural inequalities, the greater the resources in the denominator (i.e., individual and family-based resources) should be in order to establish a balance between the structural deprivations and individual resources and hence to prevent problems. The formula also clearly indicates that strengthening individuals and families has limits in efforts to get desirable developmental outcomes unless the numerator (e.g., problems in access to social and material resources) are equally addressed.

### Limitations

The present research has some limitations. The data is cross-sectional and the model tested does not imply causation between the variables. The data was collected from EA who are either college students or graduated from college. Therefore, the data is not representative of the young population in the countries involved in the present study. As addressed in the discussion, the assessment of the external developmental assets focuses on the relationships in the microsystem. Inclusion of the wider assets in the conceptualization and assessment (e.g., ways of participation and social inclusion, leisure opportunities, other cultural and social capital that helps young people to improve their perspective and skills) might reveal other developmental resources that contribute to positive sense of identity as well as other developmental outcomes. Another limitation was the exclusion of one of the external assets. The scale of constructive use of time was excluded because of its low internal consistency. A valid measure of time use would also highlight an important context of development that will shed light on positive development among emerging adults.

A final limitation was that full measurement invariance in all variables was achieved only for configural and metric invariance. Full scalar invariance was reached only in positive identity variable, for other variables there was partial invariance. Measurement invariance is a key methodological issue in comparative research (Putnick and Bornstein, 2016). However, there is no clear standards or consensus on how to proceed in case of noninvariance (Chen, 2008; Putnick and Bornstein, 2016). Research shows that partial invariance can be allowed in cases where an item is not fully noninvariant across all measurement equivalence testing steps (i.e., metric and scalar), because in such cases the analysis does not yield biased findings (Guenole and Brown, 2014). Based on this research, we proceded with mean comparisons. However, given that the field of measurement invariance testing is still unripe in providing a methodological consensus (Putnick and Bornstein, 2016), the mean comparisons across the countries in the present research should be considered with caution. Indeed, Putnick and Bornstein (2016) state that although noninvariance does not preclude further comparative analyses, it might still be indicating something important in regard to the way groups perceive the same item. The partial scalar invariance in external assets may be an indication of some cultural factors explaining the differences across countries. Such cultural factors might be related to how cultures provide external assets, that is, how they support and empower their youth and set expectations and boundaries to foster youth development. The measurement non-invariance invites revisiting of the items of developmental assets for cross-cultural measurement. Future studies might also examine different ways cultures foster the development of young members of their society.

## Conclusion and Future Research

The present results underlined the importance of supportive, empowering developmental relationships showing that despite cultural, social and economic differences, resources stemming from the developmental relationships contribute positively to positive sense of identity similarly across the four countries in the European continent. When evaluated in the light of data from other resources (e.g., Eurostat), as well as theory and critical evaluation of youth policies, the present results also underline that development of a person cannot be solely the responsibility of individuals and their families. Scales et al. (2017) state that the significance of developmental relationships and the empowering social experiences are not reflected enough in research as well as in policy and practice. Supportive and empowering relationships are crucial for development, and optimization and utilization of these relationships is possible through social policies which support families as well as provide opportunities that are beyond the power of families. Researchers who examined the youth policies which has an explicit emphasis on family also highlight this crucial issue in policy frame. Policies that focus solely on family and microenvironment to empower the youth are inadequate and that both the wider society and the state has responsibilities to facilitate successful transition to adulthood (Açıkgöz et al., 2019; Zupan, 2016). In that sense, Bronfenbrenner’s ecological systems model can serve as a useful framework to assess environmental assets and provide a template to guide both research, policymaking and its implementation.

## Data Availability Statement

The raw data supporting the conclusions of this article will be made available by the authors, without undue reservation.

## Ethics Statement

The studies involving human participants were reviewed and approved by Regional Committees for Medical and Health Research Ethics in Norway; Slovenian Research Agency; Ozyegin University Board of Research Ethics. The patients/participants provided their written informed consent to participate in this study.

## Author Contributions

All authors were involved in the design of the study and collected data in their countries. AD-G conducted the multivariate analysis of variance and drafted the manuscript. NW conducted and reported the measurement invariance and path analyses. All authors were involved in the critical review and the revision of the manuscript. All authors approved the final version of the manuscript.

## Conflict of Interest

The authors declare that the research was conducted in the absence of any commercial or financial relationships that could be construed as a potential conflict of interest.
